# Letter to the editor concerning “Development and validation of a machine learning model utilizing the ACS-NSQIP database for predicting early postoperative Re-operation in lumbar microdiscectomy” by M.M. Dagli et al.

**DOI:** 10.1016/j.bas.2026.107449

**Published:** 2026-09-21

**Authors:** Jin Yan, Yang Liu, Kaiyuan Yang, Boyan Su

**Affiliations:** a Clinical Cognitive Neuroscience, Advanced Occupational Therapy, Human Health Sciences, Graduate School of Medicine, Kyoto University, 53 Shogoin Kawahara-cho, Sakyo-ku, Kyoto, 606-8507, Japan; b Department of Neurosurgery, Beijing Tsinghua Changgung Hospital, School of Clinical Medicine, Tsinghua Medicine, Tsinghua University, No. 168 Litang Road, Changping District, Beijing, 102218, China

**Keywords:** Machine learning, Label leakage, Surgical site infection, Microdiscectomy, Prediction model

We read with interest the article by Dagli et al. (2026), which developed a machine-learning model for 30-day re-operation following surgical-site infection (SSI) after lumbar microdiscectomy and reported an area under the receiver operating characteristic curve (AUROC) of 0.996. Rare-event prediction in spine surgery is an important and under-addressed problem, and the authors’ use of nested cross-validation, Bayesian optimisation and bootstrap confidence intervals is welcome. We are concerned, however, that the reported discrimination is almost entirely recoverable from a single postoperative variable that carries information contained in the endpoint itself.

The outcome was re-operation following postoperative SSI, yet “any postoperative SSI” was retained as a predictor and was, by a wide margin, the leading SHAP feature (their Fig. 3). Table 2 of the article allows a direct check. All 462 outcome-positive patients had an SSI, whereas 597 of 79,408 controls had an SSI. Classifying every patient with an SSI as predicted to undergo re-operation therefore yields 462 true positives, 597 false positives, 78,811 true negatives and no false negatives. This one-variable rule attains an accuracy of 0.9925, specificity 0.9925, sensitivity 1.000, positive predictive value (PPV) 0.4363, negative predictive value (NPV) 1.000 and, treating binary SSI status as the score, an AUROC of 0.9962. These values reproduce the published model almost exactly (accuracy 0.993, specificity 0.993, PPV 0.436–0.437, NPV 1.000, AUROC 0.996).

Nested cross-validation, bootstrapping and SMOTE address model tuning, uncertainty and class imbalance, respectively; none restores temporal separation between predictors and the outcome. Prognostic models should be built from information available at the time the prognosis is made (Efthimiou et al., 2024), and including variables generated afterwards constitutes timing-based label leakage (Davis et al., 2024). TRIPOD + AI, to which the authors state adherence, asks that predictors be defined together with how and when they were measured relative to the intended moment of prediction (Collins et al., 2024); this timing is not reported.

The distinction determines what the model can be used for. The Discussion proposes perioperative counselling, targeted prevention and tailored follow-up, yet postoperative SSI status is by definition unavailable before an SSI has occurred. If the intended moment of prediction is instead the point of SSI diagnosis, the relevant population is the 1059 patients who developed an SSI; within that population “any SSI” is constant, and the present analysis does not demonstrate discrimination between the 462 patients who underwent re-operation and the 597 who did not.

A leakage-free reanalysis is possible using the existing data. For a perioperative prediction target, the model could be refitted using only variables available by the end of the index operation, with cross-validated discrimination, precision–recall performance and calibration reported. For prediction at SSI diagnosis, the analysis could instead be restricted to infected patients and exclude variables downstream of that point. Either analysis would offer a clinically interpretable contribution to rare-event modelling in spine surgery. Until leakage-excluded performance is reported, we would caution against interpreting an AUROC of 0.996 as evidence of a clinically deployable perioperative risk-stratification tool.

## Declaration of generative AI and AI-assisted technologies in the manuscript preparation process

During the preparation of this work, the authors used OpenAI Codex solely to improve the readability and language of the manuscript. After using this tool, the authors reviewed and edited the content as needed and take full responsibility for the content of the published article.

## Ethical considerations

Not applicable. This Letter reports no original research involving human participants or animals; all data discussed are contained in the published article by Dagli et al. (2026).

## Author contributions

J.Y. and Y.L.: conception of the Letter, audit of the published source data, quantitative reconstruction, manuscript drafting, manuscript revision, and approval of submission. K.Y.: independent verification of the source data and calculations, methodological review, manuscript revision, and approval of submission. B.S.: supervision, critical revision of the manuscript, and approval of submission. All authors approved the final version and agree to be accountable for the work.

## Data availability

No new data were generated. All values used in this Letter are taken from Tables 2 and 3 of the article by Dagli et al. (2026).

## Funding

This work received no specific grant from any funding agency in the public, commercial, or not-for-profit sectors.

## Declaration of competing interest

The authors declare that they have no known competing financial interests or personal relationships that could have appeared to influence the work reported in this paper.
